# MDA-SKF: Similarity Kernel Fusion for Accurately Discovering miRNA-Disease Association

**DOI:** 10.3389/fgene.2018.00618

**Published:** 2018-12-10

**Authors:** Limin Jiang, Yijie Ding, Jijun Tang, Fei Guo

**Affiliations:** ^1^School of Computer Science and Technology, College of Intelligence and Computing, Tianjin University, Tianjin, China; ^2^School of Electronic and Information Engineering, Suzhou University of Science and Technology, Suzhou, China; ^3^Department of Computer Science and Engineering, University of South Carolina, Columbia, SC, United States

**Keywords:** Laplacian Regularized Least Squares, disease similarity, miRNA similarity, miRNA-disease association, Similarity Kernel Fusion

## Abstract

Identifying accurate associations between miRNAs and diseases is beneficial for diagnosis and treatment of human diseases. It is especially important to develop an efficient method to detect the association between miRNA and disease. Traditional experimental method has high precision, but its process is complicated and time-consuming. Various computational methods have been developed to uncover potential associations based on an assumption that similar miRNAs are always related to similar diseases. In this paper, we propose an accurate method, MDA-SKF, to uncover potential miRNA-disease associations. We first extract three miRNA similarity kernels (miRNA functional similarity, miRNA sequence similarity, Hamming profile similarity for miRNA) and three disease similarity kernels (disease semantic similarity, disease functional similarity, Hamming profile similarity for disease) in two subspaces, respectively. Then, due to limitations that some initial information may be lost in the process and some noises may be exist in integrated similarity kernel, we propose a novel Similarity Kernel Fusion (SKF) method to integrate multiple similarity kernels. Finally, we utilize the Laplacian Regularized Least Squares (LapRLS) method on the integrated kernel to find potential associations. MDA-SKF is evaluated by three evaluation methods, including global leave-one-out cross validation (LOOCV) and local LOOCV and 5-fold cross validation (CV), and achieves AUCs of 0.9576, 0.8356, and 0.9557, respectively. Compared with existing seven methods, MDA-SKF has outstanding performance on global LOOCV and 5-fold. We also test case studies to further analyze the performance of MDA-SKF on 32 diseases. Furthermore, 3200 candidate associations are obtained and a majority of them can be confirmed. It demonstrates that MDA-SKF is an accurate and efficient computational tool for guiding traditional experiments.

## 1. Introduction

MicroRNAs (miRNAs) are a set of small non-coding RNAs (about 20−25 nucleotides) that can normally function as negative regulators of target messenger RNA (mRNA) expression in the process of post-transcription (Jiang et al., 2010b). They restrain target mRNA via base pairing, and influence gene translation. And, it has been verified that miRNA also function as positive regulators (Lu et al., 2008). In recent years, some existing works demonstrate that miRNAs are involved in many significant biologic processes, including cell differentiation, development, proliferation, and signal transduction (Carthew and Sontheimer, 2009). In addition, some previous studies prove that miRNAs are related to various diseases, including cancers (Iorio et al., 2005), Alzheimer (Cogswell et al., 2008), Diabetes (Caporali et al., 2011), and Lymphoma (Roehle et al., 2008). For example, the expression level of hsa-mir-21 is related to more than 125 diseases (Li et al., 2014). Therefore, identifying more associations between miRNAs and diseases is beneficial for diagnosis and treatment of human complex diseases.

Traditional experimental method has high precision for discovering potential associations, but its process is complicated and time-consuming. It is especially important to develop an efficient and convenient method to detect the association between miRNA and disease. Up to now, massive associations are obtained via traditional experiments and stored in some public database. The dbDEMC (Yang et al., 2010) collects 20037 associations including 2,224 miRNAs and 36 cancer types. The HMDD (Li et al., 2014) stores 10,368 miRNA-disease associations including 572 miRNAs and 378 diseases. The miR2Disease (Jiang et al., 2009) stores 3,273 miRNA-disease associations including 349 miRNAs and 163 diseases. Based on known associations, various computational methods have been developed to uncover potential associations.

In the past few years, computational methods achieve outstanding performance for discovering the novel associations between miRNAs and diseases (Lan et al., 2016; Zeng et al., 2016b; Zou et al., 2016; Chen et al., 2017a; Li et al., 2017b). Most of existing computational methods are based on an assumption that miRNAs with high similarity tend to be related with same diseases and vice versa (Liu et al., 2016). The method proposed by Jiang et al. (2010a) uses a discrete hyper-geometric probability distribution to calculate the strength of miRNA-disease associations. The HDMP (Xuan et al., 2013) calculates the miRNAs functional similarity that be assigned different weights on the basis of miRNA family and cluster. Then, all the unlabeled miRNAs are ranked by their final scores. The RWRMDA (Chen et al., 2012) uses miRNAs functional similarity network and the model of Random Walk to calculate the probability of candidate miRNAs for a special disease. The MIDP (Xuan et al., 2015) employs an improved Random Walk to set scores for candidate miRNAs, so the miRNA with larger score has higher possibility associated with the special disease.

Above methods have significant performances at the aspect of finding novel associations, but can not work for a new disease without known related miRNAs. The WBSMDA (Chen et al., 2016) uses miRNA functional similarity matrix and disease semantic similarity matrix and Gaussian interaction profile kernel similarity matrix to reconstruct miRNA and disease similarity matrix. Then, an probability value for the miRNA-disease association can be calculate by using Within-Scores and Between-Scores. The WBSMDA solves the limitation of previous computational models, that is to say, it could work for diseases without any known related miRNAs and miRNAs without any known associated diseases. The NCPMDA (Gu et al., 2016) reconstructs miRNA similarity matrix by using miRNA functional similarities, miRNA family information and known associations, and constructs disease similarity matrix by integrating disease semantic similarity matrix and known associations. Then, the network consistency projection is employed to calculate final score of miRNA-disease pair. This method gets outstanding performance when handling a disease without any known related miRNAs.

Recently, machine learning algorithms are popular methods for identifying miRNA-disease associations (Luo and Xiao, 2017; Xiao et al., 2017; Luo et al., 2018). RLSMDA (Chen and Yan, 2014) constructs miRNA functional similarity and disease semantic similarity in two different subspaces. Then, two cost functions are constructed by Regularized Least Squares respectively. Finally, all predicted associations between two subspaces are combined to denote as the final results. This method has excellent performance at the aspect of uncovering potential associations between miRNAs and diseases. The PBMDA (You et al., 2017) uses miRNA functional similarity, disease semantic similarity, Gaussian interaction profile kernel similarity and known associations to construct a heterogeneous graph. A specific depth-first search algorithm is employed to traverse all pathes in the graph. Finally, the miRNA-disease score can be obtained to represent association probability. The LRSSLMDA (Chen and Huang, 2017) extracts miRNA functional similarity, disease semantic similarity, Gaussian interaction profile kernel similarity, and applies the Laplacian Regularized Sparse Subspace Learning to discover potential associations between miRNAs and diseases. The method proposed by Zeng et al. (2018) constructs a bilayer network by integrating miRNA and disease similarity networks and adjacency network. Then, this bilayer network and structural perturbation method (SPM) are employed to uncover potential associations.

Although all the mentioned methods have achieved outstanding performance for uncovering potential associations, most of them have suffered from different limitations or restrictions (Chen et al., 2017c; Peng et al., 2018). For example, how better to integrate these multiple kernels when extracting various similarity kernels for miRNAs and diseases. Most of models employ the linear weighting method to integrate multiple kernels into one kernel (Chen et al., 2017b; Lan et al., 2017). We believe that some information may be lost in the process and noises may exist in the final similarity kernel for Similarity Network Fusion (SNF) (Wang et al., 2014). Therefore, we propose the method of Similarity Kernel Fusion (SKF) in this paper. We retain the initial information of each kernel when integrating multiple kernels, and use a weight matrix to eliminate noises in the integrated similarity kernel.

In this paper, we introduce the method of MDA-SKF to uncovering potential associations between miRNAs and diseases. First, we construct similarity kernels from two subspaces, including miRNA subspace and disease subspace. In miRNA subspace, we extract miRNA functional similarity kernel and miRNA sequence similarity kernel. And we first propose miRNA Hamming profile similarity kernel using the miRNA-disease associations. These similarity kernels are used to represent miRNA similarity. In disease subspace, we extract disease semantic similarity kernel and disease functional similarity kernel. And we first propose disease Hamming profile similarity kernel by using disease-miRNA associations. These similarity kernels are employed to represent disease similarity. Second, we respectively integrate three kernels into one kernel by using SKF in each subspace. Then, we use the Laplacian Regularized Least Squares (LapRLS) (Xia et al., 2010) and integrated kernel to uncover potential associations in two subspaces. Finally, we average two predicted association matrices as the final predicted associations.

Three evaluation methods are used to verify the performance of MDA-SKF, including global Leave-One-Out Cross Validation (global LOOCV), local Leave-One-Out Cross Validation (local LOOCV), and 5-fold cross validation (5-fold CV). Compared with existing seven methods, MDA-SKF has the outstanding performance for uncovering potential miRNA-disease associations. For further verification, we use global validation and local validation to analyze 32 diseases associations. The experimental results show that our method have reliable performance on detecting novel associations. Meanwhile, we find that some special associations and corresponding miRNAs require more attention. These associations can be used to guide the traditional experience.

## 2. Materials and Methods

**Figure 1:**
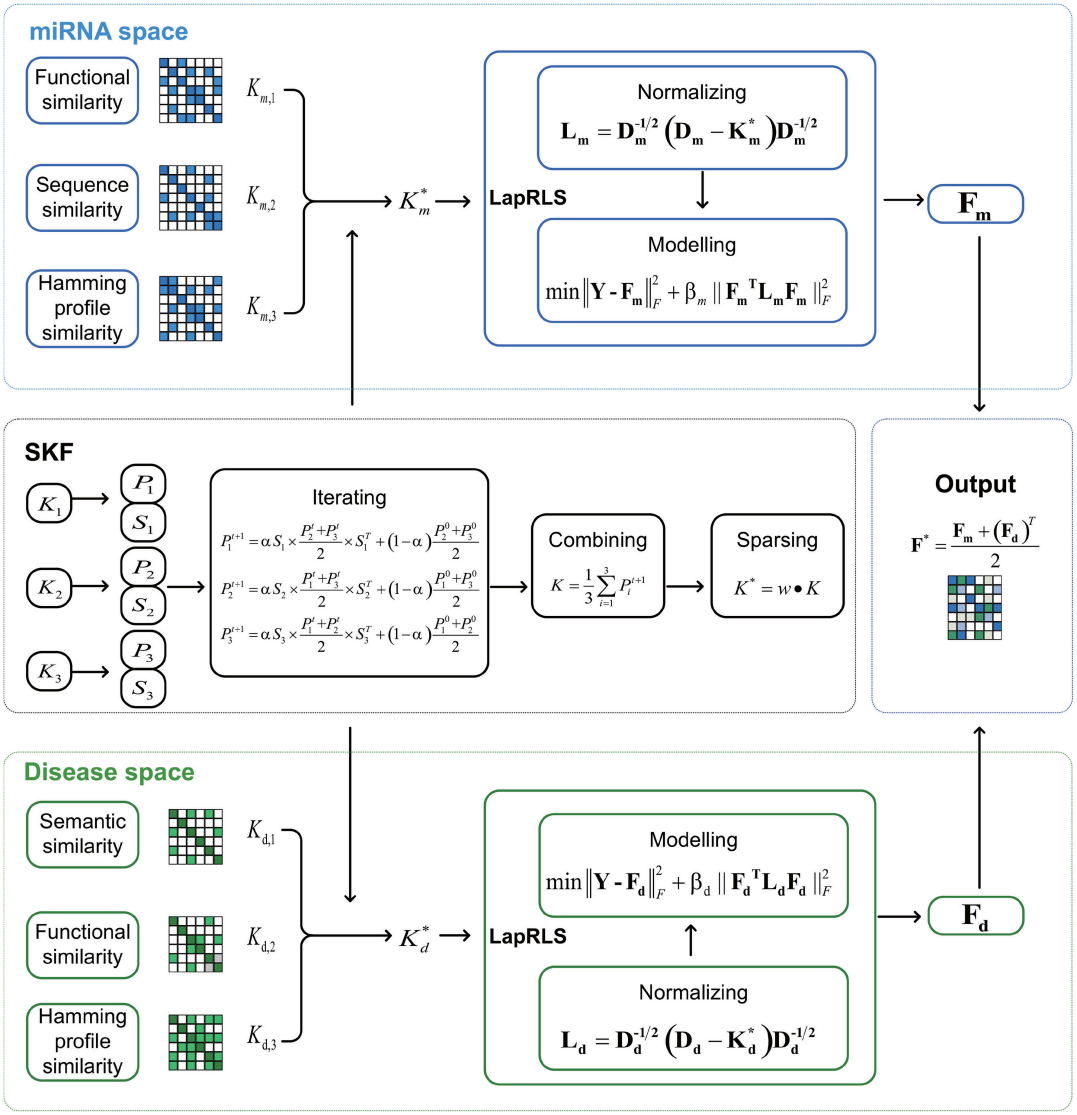
The flowchart of MDA-SKF for uncovering miRNA-disease associations.

In this paper, we respectively establish three miRNA similarity kernels and three disease similarity kernels to predict association between miRNA and disease. Firstly, we integrate these kernels into one miRNA kernel and one disease kernel using the method of Similarity Kernel Fusion (SKF). Then, we employ Laplacian Regularized Least Squares on the integrated kernels to uncover potential association. Finally, we combine two predicted adjacency matrices from miRNA and disease subspaces to analyze potential associations. The flow chart of SKFMDA is shown in Figure 1.

### 2.1. Human miRNA-Disease Association Dataset

We get 5,430 miRNA-disease associations including 495 miRNAs and 383 diseases, which are downloaded from HMDD (Li et al., 2014) database. The set of miRNAs is denoted by $M={\left\{m_{i}\right\}}_{i=1}^{p}$ and the set of diseases is denoted by $D={\left\{d_{j}\right\}}_{j=1}^{q}$. The association matrix is represented by *Y*∈*R*^*p*×*q*^, where *Y*(*i, j*)∈{0, 1}. When the miRNA *m*_*i*_ is association with the disease *d*_*j*_, *Y*(*i, j*) is set to 1; otherwise, *Y*(*i, j*) is set to 0.

### 2.2. Similarity Kernels for Diseases and miRNAs

Our method is based on the assumption that miRNAs with high similarity apt to be related with the same diseases and diseases with high similarity apt to be related with the same miRNA. Therefore, we respectively establish three miRNA similarity kernels and three disease similarity kernels to uncover potential association between miRNA and disease.

#### 2.2.1. Disease Semantic Similarity

In the MeSH (Lowe and Barnett, 1994) database, the disease *d*_*i*_ can be marked as a node in Directed Acyclic Graph (DAG). We denote a subnetwork as *G*_*d*_*i*__ = (*d*_*i*_, *T*_*d*_*i*__, *E*_*d*_*i*__), where *T*_*d*_*i*__ is the set of all ancestor nodes of *d*_*i*_ including itself and *E*_*d*_*i*__ is the set of corresponding links. A semantic score of each disease can be calculated by Equation (1) (Wang et al., 2010).

(1) $$D_{d_{i}}(t)=\{\begin{array}{ll} 1 & if\;t=d_{i}\\ max\left\{\Delta *D_{d_{i}}\left(t^{\prime }\right)|t^{\prime }\in \;children\;of\;t\right\} & if\;t\neq d_{i} \end{array}$$

where the disease *t*∈*T*_*d*_*i*__; Δ is the semantic contribution factor and Δ = 0.5.

Also, we denote the semantic score of the disease *d*_*i*_ by Equation (2).

(2) $$\begin{array}{r} DV\left(d_{i}\right)=\sum_{t\in T_{d_{i}}}D_{d_{i}}\left(t\right) \end{array}$$

Then, we calculate the disease semantic similarity value between *d*_*i*_ and *d*_*j*_ by Equation (3).

(3) $$\begin{array}{r} K_{d,1}\left(d_{i},d_{j}\right)=\frac{\sum_{t\in T_{d_{i}}\cap T_{d_{j}}}\left(D_{d_{i}}\left(t\right)+D_{d_{j}}\left(t\right)\right)}{DV\left(d_{i}\right)+DV\left(d_{j}\right)} \end{array}$$

Finally, we obtain the disease semantic similarity $K_{d,1}\in R^{q\times q}$.

#### 2.2.2. Disease Functional Similarity

In the previous works (Luo et al., 2017), the associations between diseases and genes are used to calculate disease functional similarity. We download the Log Likehood Score (LLS) that is the probability of a functional linkage between genes in the HumanNet (Lee et al., 2011) database. We normalize the LLS by Equation (4).

(4) $$\begin{array}{r} LLS^{*}\left(g_{k},g_{s}\right)=\frac{LLS\left(g_{k},g_{s}\right)-LLS_{min}}{LLS_{max}-LLS_{min}} \end{array}$$

where *LLS*(*g*_*k*_, *g*_*s*_) is the LLS between *k*-th and *s*-th genes; $LLS^{*}\left(g_{k},g_{s}\right)$ is the normalized LLS score; *LLS*_*min*_ and *LLS*_*max*_ represent the minimum and maximum LLS scores in HumanNet, respectively.

We define the functional similarity score between genes by Equation (5).

(5) $$FS\left(g_{k},g_{s}\right)=\{\begin{array}{lll} 1 & \; & \;if\;k=s\\ LLS^{*}\left(g_{k},g_{s}\right) & \; & if\;k\neq s\cap \;e(k,s)\in S_{HumanNET}\\ 0 & \; & if\;k\neq s\cap e(k,s)\notin S_{HumanNET} \end{array}$$

where *S*_*HumanNET*_ is the set of all links between genes in the HumanNet database; *e*(*k, s*) is the link between *k*-th and *s*-th genes.

Then, we define the functional similarity score between a gene *g* and a set of genes *G* as Equation (6).

(6) $$\begin{array}{r} F_{G}\left(g\right)=\underset{g_{s}\in G}{\max}FS\left(g,g_{s}\right) \end{array}$$

The associations between diseases and genes are downloaded from SIDD (Liang et al., 2013). We define the functional similarity score between diseases by Equation (7).

(7) $$\begin{array}{r} K_{d,2}\left(d_{i},d_{j}\right)=\frac{\underset{g_{k}\in G_{j}}{\sum }F_{G_{i}}\left(g_{k}\right)+\underset{g_{s}\in G_{i}}{\sum }F_{G_{j}}\left(g_{s}\right)}{\left|G_{i}|+|G_{j}\right|} \end{array}$$

where *g*_*k*_∈*G*_*j*_ and *g*_*s*_∈*G*_*i*_; *G*_*i*_ and *G*_*j*_ represent sets of genes which are related to diseases *d*_*i*_ and *d*_*j*_, respectively.

Finally, we obtain the disease functional similarity $K_{d,2}\in R^{q\times q}$.

#### 2.2.3. MiRNA Functional Similarity

We construct miRNA functional similarity kernel $K_{m,1}\in R^{p\times p}$, according to MISIM (Wang et al., 2010) proposed by Wang et al. This method used the disease semantic similarity and the known associations between miRNAs and diseases to structure miRNA functional similarity kernel. Here, *K*_*m*, 1_(*m*_*i*_, *m*_*j*_) is the functional similarity score between miRNAs *m*_*i*_ and *m*_*j*_.

#### 2.2.4. MiRNA Sequence Similarity

We obtain 495 miRNA sequences from miRBase database(Kozomara and Griffithsjones, 2014), and calculate sequence similarity of miRNAs by using the Needleman–Wunsch Algorithm. Then, we obtain miRNA sequence similarity kernel $K_{m,2}\in R^{p\times p}$, where *K*_*m*, 2_(*m*_*i*_, *m*_*j*_) is the sequence similarity score between miRNAs *m*_*i*_ and *m*_*j*_.

#### 2.2.5. Hamming Profile Similarity

The assumption that similar diseases are always related to similar miRNAs, is employed to uncover miRNA-disease associations. For a pair of vectors whose lengths are same, Hamming profile is the number of elements of which corresponding values are different. Higher Hamming profile value indicates lower similarity for two vectors. Therefore, we use Hamming profile and the topologic information of all known associations to measure disease similarity. Here, Hamming profile similarity kernel for diseases is defined as Equation (8).

(8) $$\begin{array}{r} K_{d,3}\left(d_{i},d_{j}\right)=1-\frac{\left|IP\left(d_{i}\right)!=IP\left(d_{j}\right)\right|}{\left|IP\left(d_{i}\right)\right|} \end{array}$$

where $K_{d,3}\in R^{q\times q}$ is the Hamming profile similarity for diseases; $IP\left(d_{i}\right)\in {\left\{0,1\right\}}^{p\times 1}$ is the *i*-th column of the association matrix *Y*.

Similarly, we calculate Hamming profile similarity kernel for miRNAs as Equation (9).

(9) $$\begin{array}{r} K_{m,3}\left(m_{i},m_{j}\right)=1-\frac{\left|IP\left(m_{i}\right)!=IP\left(m_{j}\right)\right|}{\left|IP\left(m_{i}\right)\right|} \end{array}$$

where $K_{m,3}\in R^{p\times p}$ is the Hamming profile similarity for miRNAs; $IP\left(m_{i}\right)\in {\left\{0,1\right\}}^{1\times q}$ denotes the *i*-th row of the associations matrix *Y*.

### 2.3. Similarity Kernel Fusion

We extract three miRNA similarity kernels (miRNA functional similarity, miRNA sequence similarity, Hamming profile similarity for miRNA) and three disease similarity kernels (disease semantic similarity, disease functional similarity, Hamming profile similarity for disease) in the above section.

In the following, we use similarity kernel fusion (SKF) to integrate three miRNA similarity kernels *K*_*m, l*_, *l* = 1, 2, 3. Therefore, we get the integrated similarity kernel $K_{m}^{*}\in R^{p\times p}$.

Firstly, we normalize each original kernel by Equation (10).

(10) $$\begin{array}{r} P_{m,l}\left(m_{i},m_{j}\right)=\frac{K_{m,l}\left(m_{i},m_{j}\right)}{\underset{m_{k}\in M}{\sum }K_{m,l}\left(m_{k},m_{j}\right)} \end{array}$$

where *P*_*m, l*_ represents a normalized kernel and satisfies $\underset{m_{k}\in M}{\sum }P_{m,l}\left(m_{k},m_{j}\right)=1$.

Secondly, we construct a sparse kernel for each original kernel by Equation (11).

(11) $$S_{m,l}\left(m_{i},m_{j}\right)=\{\begin{array}{ll} 0 & \;if\;m_{j}\notin N_{i}\\ \frac{K_{m,l}\left(m_{i},m_{j}\right)}{\sum_{m_{k}\in N_{i}}K_{m,l}\left(m_{i},m_{k}\right)} & \;if\;m_{j}\in N_{i} \end{array}$$

where *S*_*m, l*_ represents a sparse kernel and satisfies $\underset{m_{j}\in M}{\sum }S_{m,l}\left(m_{i},m_{j}\right)=1$; *N*_*i*_ represents a set of all neighbors of *m*_*i*_ including itself.

Thirdly, we integrate three miRNA kernels by Equation (12).

(12) $$\begin{array}{r} P_{m,l}^{t+1}=\alpha \left(S_{m,l}\times \frac{\underset{r\neq l}{\sum }P_{m,r}^{t}}{2}\times S_{m,l}^{T}\right)+\left(1-\alpha \right)\left(\frac{\underset{r\neq l}{\sum }P_{m,r}^{0}}{2}\right) \end{array}$$

where $P_{m,r}^{0}$ represents the initial status of *P*_*m, r*_; $P_{m,l}^{t+1}$ is the status of *l*-th kernel after *t*+1 iterations; α∈(0, 1).

After *t*+1 iterations, the overall kernel can be computed as Equation (13).

(13) $$\begin{array}{r} K_{m}=\frac{1}{3}\overset{3}{\underset{l=1}{\sum }}P_{m,l}^{t+1} \end{array}$$

Finally, a weight matrix is established to further eliminate noise in the overall kernel as Equation (14).

(14) $$w_{m}\left(m_{i},m_{j}\right)=\{\begin{array}{ll} 1 & \;if\;m_{j}\in N_{i}\cap m_{i}\in N_{j}\\ 0 & \;if\;m_{j}\notin N_{i}\cap m_{i}\notin N_{j}\\ 0.5 & \;otherwise\; \end{array}$$

The integrated miRNA similarity kernel can be obtained as Equation (15).

(15) $$\begin{array}{r} K_{m}^{*}=w_{m}\circ K_{m} \end{array}$$

Similarity, we calculate the integrated disease similarity kernel as $K_{d}^{*}\in R^{q\times q}$.

### 2.4. Laplacian Regularized Least Squares

In this paper, we use Laplacian Regularized Least Squares (LapRLS) to uncover potential miRNA-disease associations. For the miRNA subspace, The objective function of LapRLS is defined as Equation (16).

(16) $$\begin{array}{r} \underset{F_{m}}{\min}\;||Y-F_{m}|{|}_{F}^{2}+\beta_{m}||F_{m}^{T}L_{m}F_{m}|{|}_{F}^{2} \end{array}$$

where *Y* is the known association matrix; β_*m*_ is the regularization coefficient of LapRLS. $F_{m}\in R^{p\times q}$ represents the predicted association matrix in the miRNA subspace; $L_{m}=D_{m}^{-\frac{1}{2}}\left(D_{m}-K_{m}^{*}\right)D_{m}^{-\frac{1}{2}}$, in which *D*_*m*_ is a diagonal matrix whose diagonal element is the sum of the row elements of $K_{m}^{*}$.

The derivation of optimization algorithm were presented in Xia et al. (2010). We calculate the predicted association matrix $F_{m}\in R^{p\times q}$ in the miRNA subspace as Equation (17).

(17) $$\begin{array}{r} F_{m}=K_{m}^{*}{\left(K_{m}^{*}+\beta_{m}L_{m}K_{m}^{*}\right)}^{-1}Y \end{array}$$

Similarity, we can calculate the predicted association matrix $F_{d}\in R^{q\times p}$ in the disease subspace as Equation (18).

(18) $$\begin{array}{r} F_{d}=K_{d}^{*}{\left(K_{d}^{*}+\beta_{d}L_{d}K_{d}^{*}\right)}^{-1}Y^{T} \end{array}$$

The predicted matrices in miRNA and disease subspaces are *F*_*m*_ and *F*_*d*_, respectively. Then, we define the final predicted association matrix as Equation (19).

(19) $$\begin{array}{r} F^{*}=\frac{F_{m}+F_{d}^{T}}{2} \end{array}$$

where F^*^∈*R*^*p*×*q*^.

## 3. Results

In this section, we analyze the performance of MDA-SKF from many aspects. First, we introduce three evaluation methods (global LOOCV, local LOOCV, and 5-fold CV) and two validation methods (global verification and local verification) to analyze the performance of MDA-SKF. Second, we discuss about the convergence and the parameter selection of SKF. Third, we compare the performance of SKF with SNF and average kernel. Fourth, we compare the performance of MDA-SKF with other excellent methods for uncovering potential associations between miRNAs and diseases. Fifth, we use case studies to further evaluate the reliability of MDA-SKF.

### 3.1. Evaluation Criteria and Verification Methods

In this paper, we use two evaluation criteria including Area Under the Curve (AUC) and Area Under the Precision-Recall curve (AUPR) to evaluate the performance of models. AUC is the area under the receiver operating characteristic (ROC) curve, which is created by plotting true positive rate against false positive rate at various threshold settings. AUPR is the area under the curve that is created by plotting precision against recall at various threshold settings.

In the process of experiments, global LOOCV, local LOOCV, and 5-fold CV are applied to evaluate the model's performance. In the global LOOCV, one of 5,430 known associations is left out in turn as the test set, and other associations are remained as the training set. In the local LOOCV, the known associations between a special disease and all miRNAs are left out as the test set, and other associations are regarded as training set. In the 5-fold, all known associations are randomly divided into five non-overlapping sets. each set is employed in turn to as test set and other sets are employed to as training set. In the process of experiments, the known associations in test set are reset to unknown, that is to say, some 1 are replaced by 0 in the association matrix *Y*.

Massive associations between miRNAs and diseases are obtained via the traditional experiment and stored in several databases, which provide a good condition for evaluating the performance of MDA-SKF. We use two methods including global validation and local validation to further analyze the reliability of MDA-SKF. In the global validation, we regard 5,430 known associations as training set that is used to uncover potential associations. These candidate associations are confirmed by the miR2Disease and dbDEMC databases. In the local validation, all known associations that are related to a special disease are reset to unknown ones. We use the rest of association as training set to uncover potential associations for this special disease. These candidate associations are confirmed by the HMDD, miR2Disease, and dbDEMC databases.

### 3.2. Convergence Performance

**Figure 2:**
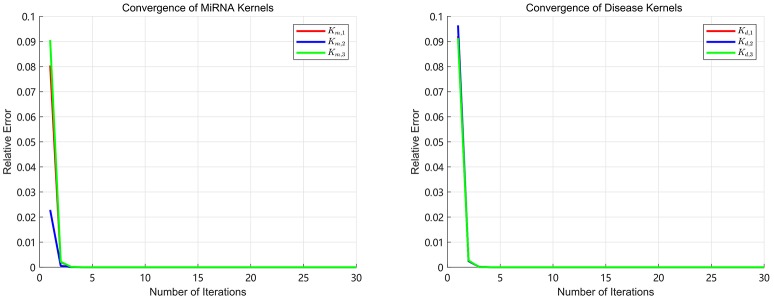
The relative errors of SKF model with different number of iterations.

Since the convergence is very important for an iterative algorithm, we analyze the number of iterations of SKF. We define the relative error as $E_{t}=\frac{\left||P_{t+1}-P_{t}|\right|}{\left||P_{t}|\right|}$ in the process of iterations. We turn the number of iterations from 1 to 30 with step 1 to calculate the *E* after each iteration. The convergence processes of three miRNA kernels and three disease kernels are calculated in our experiments and the results of *E* are shown in Figure 2. It can be clearly seen that the process of convergence is very fast and the value of *E* achieves to 10^−7^ after 5 iterations. This phenomenon demonstrates that SKF model have excellent convergence performance in the process of integrating multiple kernels. In this paper, we set the number of iterations as 10 to ensure that it is enough to converge.

### 3.3. Parameter Selection

**Figure 3:**
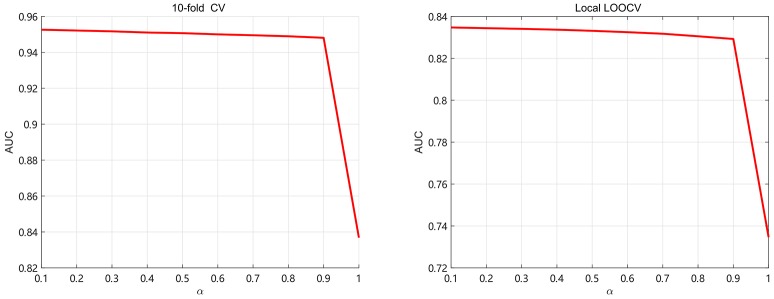
The AUC values of SKF model with different values of α.

In this section, we discuss about the parameter selection of SKF. There are two parameters α and the size of neighbors denoted as *k*. For selecting parameter α ,we use 5-fold CV and local LOOCV to analyze the values of α. We take α from 0.1 to 1 with step 0.1 in order to calculate AUC, shown in Figure 3. It can be found that AUC keep little fluctuation in the range between 0.1 and 0.9. As we can see, the value of AUC decreases by at least 0.1 when α = 1 (removing the original kernel information). It demonstrates that retaining the original information of each kernel is significant for integrating multiple kernels. In this paper, the value of α is set to 0.1.

**Figure 4:**
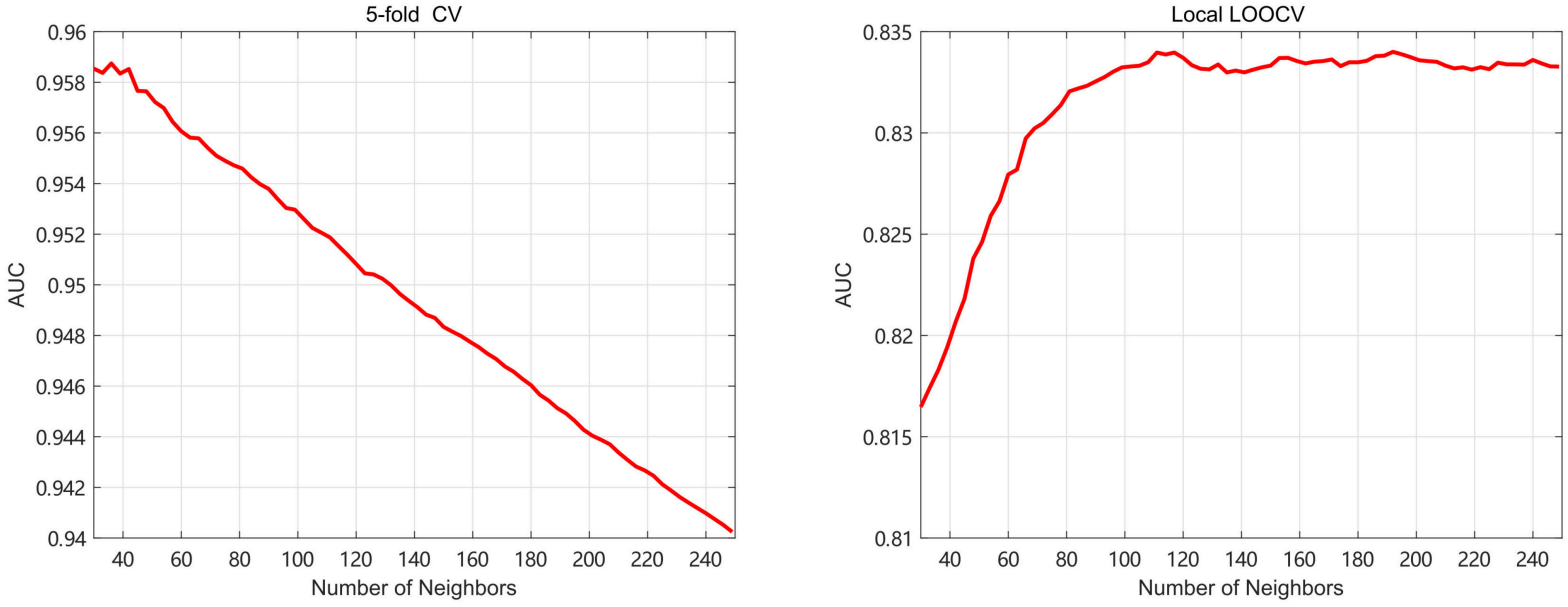
The AUC values of SKF model with different numbers of neighbors.

Meanwhile, the number of neighbors is an important parameter in this paper. It is related to the amount of important information and the noise reduction. In the 5-fold, *k* is taken from 30 to 100 with step 3 to find the optimal value. In the local LOOCV, the *k* is gradually varying from 30 to 350 with step 3 to find the best value. In Figure 4, we select the optimal *k* by the highest AUC value, and find that 36 and 192 are the best parameters of *k* for 5-fold and local LOOCV, respectively. Since both global LOOCV and 5-fold are similar, *k* is set to 36 in the global LOOCV. It's obvious that the value of *k* in the local LOOCV is bigger than that in the 5-fold. In the local LOOCV, our method produces the novel disease without known miRNA-based associations, so needs much more information about miRNA and disease similarity kernels.

**Figure 5:**
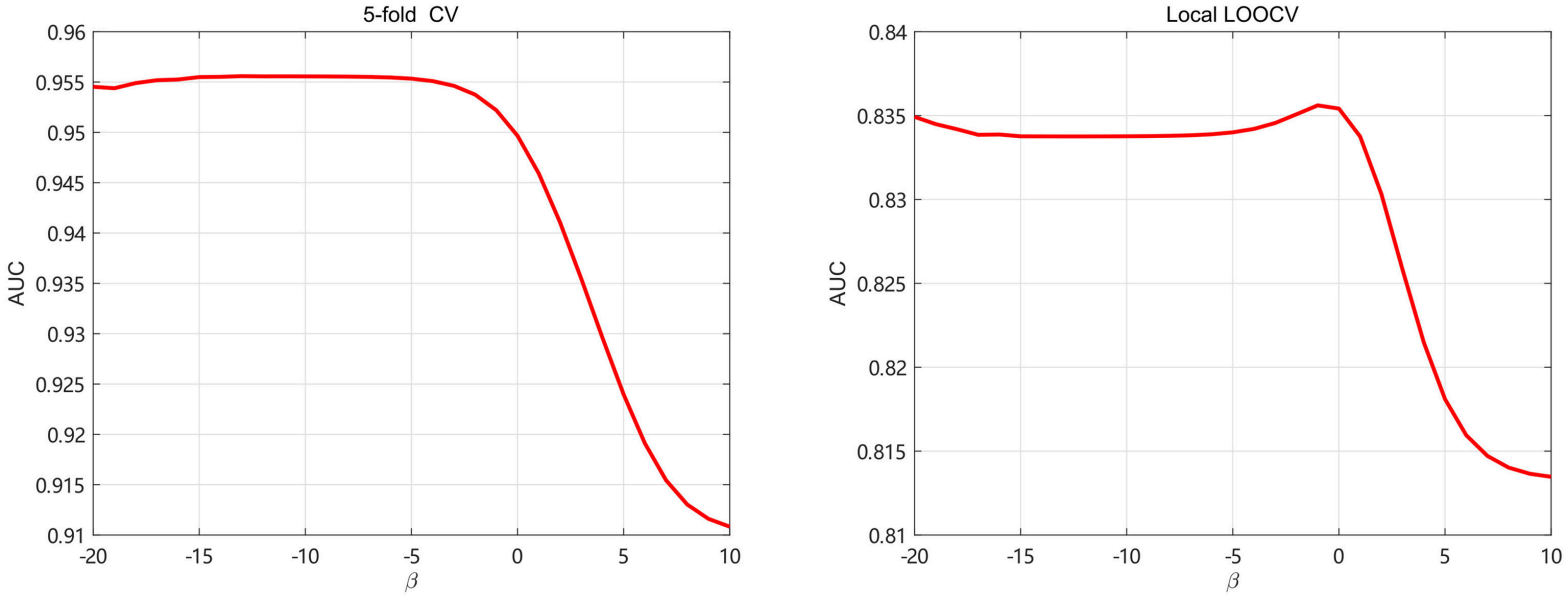
The AUC values of SKF model with different values of β.

The regularization coefficients of LapRLS, β_*m*_ and β_*d*_, are closely related to the performance of LapRLS. We make β_*m*_ equal to β_*d*_ in this paper. To get obtain the optimal β, we take β from 2^−20^ to 2^10^ and use 5-fold CV and local LOOCV to analyze the performance of LapRLS with different values of β. The results are shown in Figure 5. As seen in Figure 5, the AUC decreases when β increases from 2^0^ to 2^10^ and keeps slight change when β less than 2^−3^ and 2^0^ for 5-fold CV and local LOOCV, respectively. In the 5-fold CV, the best AUC is 0.9553 when β are 2^−5^. In the local LOOCV, the best AUC is 0.8356 when β is 2^−1^. Therefore, we select the optimal β as 2^−5^ and 2^−1^ for 5-fold CV and local LOOCV, respectively.

### 3.4. Comparison With Other Fusion Strategies

**Figure 6:**
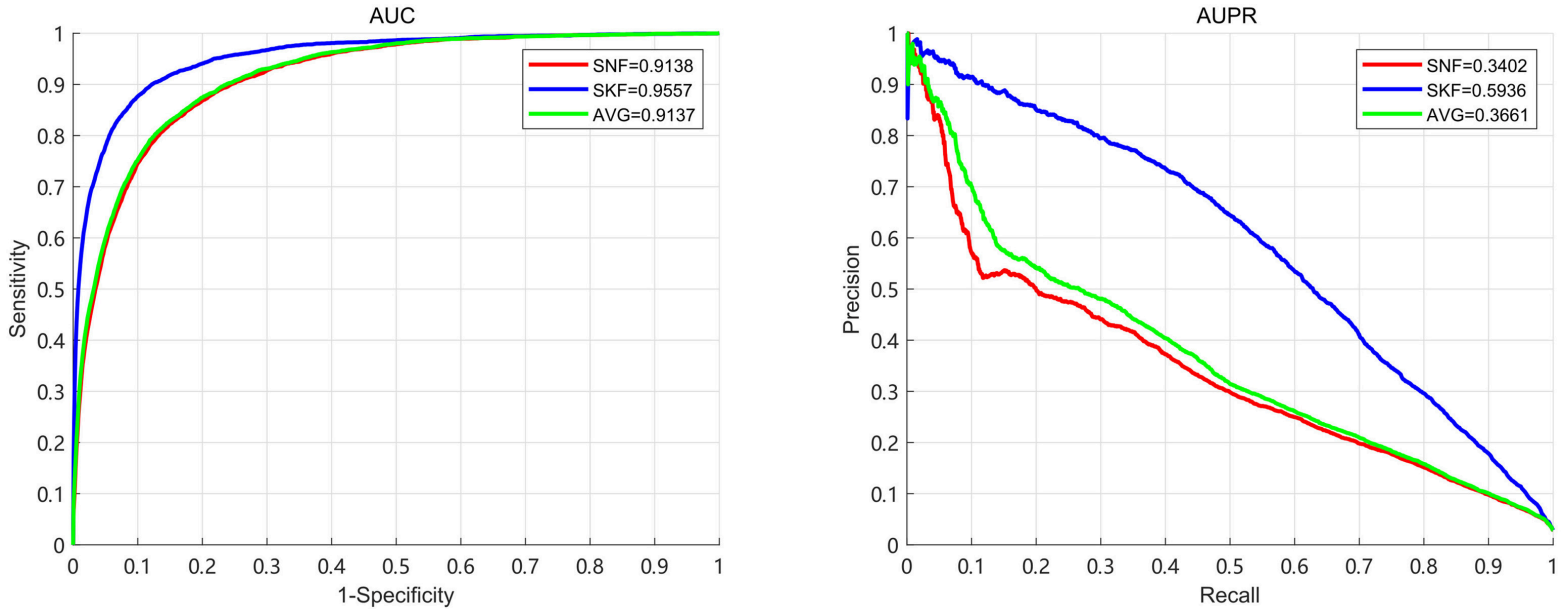
The AUC and AUPR of three fusion strategies in the 5-fold CV.

In this section, we compare the performance of Similarity Kernel Fusion (SKF) with Similarity Network Fusion (SNF) and average kernel fusion (AVG). The results demonstrate that SKF have significant performance in integrating multiple kernels. We use 5-fold CV to evaluate the performance of three fusion strategies. The results are shown in Figure 6. It can be observed that the best AUC of 0.9520 and the best AUPR of 0.5689 are obtained by SKF. Comparing with SNF, SKF achieves AUC improvement of 0.037 (0.9520 over 0.9150) and AUPR improvement of 0.2247 (0.5689 over 0.3442). Comparing with AVG, SKF achieves AUC improvement of 0.0268 (0.9520 over 0.9252) and AUPR improvement of 0.1458 (0.5689 over 0.4231). It shows that SKF is more excellent than SNF at the aspect of uncovering associations between miRNAs and diseases.

### 3.5. Comparison With Other Existing Methods

In this section, we compare the prediction performance of MDA-SKF with other seven existing methods [i.e., PBMDA (You et al., 2017), MCMDA (Li et al., 2017a), NCPMDA (Gu et al., 2016), WBSMDA (Chen et al., 2016), HDMP (Xuan et al., 2013), RLSMDA (Chen and Yan, 2014), and LRSSLMDA (Chen and Huang, 2017)] in global LOOCV, local LOOCV and 5-fold CV. Because other existing methods employ 5-fold CV in their paper, we choose 5-fold CV rather than 5-fold CV in this section. In Table  1, MDA-SKF obtains the highest AUCs in 5-fold CV (0.9501) and global LOOCV (0.9536), but NCPMDA obtains the best AUC (0.8584) in local LOOCV. Comparing with other existing methods, MDA-SKF achieves AUC improvement of at least 0.0358 and 0.0316 in global LOOCV and 5-fold CV, respectively.

**Table 1:** The comparison results between SKFMDA and other seven computational methods.

**Methods**	**Global LOOCV**	**Local LOOCV**	**5-fold CV**
PBMDA	0.9169	0.8341	0.9172
MCMDA	0.8749	0.7718	0.8767
NCPMDA	0.9073	**0.8584**	0.8763
WBSMDA	0.8030	0.8031	0.8185
HDMP	0.8366	0.7702	0.8342
RLSMDA	0.8426	0.6953	0.8569
LRSSLMDA	0.9178	0.8418	0.9181
**MDA-SKF**	**0.9576**	0.8356	**0.9557**

*Bold values represent the best value in columns*.

### 3.6. Case Studies

In this section, we employ global validation and local validation on multiple important human diseases to further evaluate the reliability of MDA-SKF. To evaluate the performance of MDA-SKF, we select 32 diseases associated with more miRNAs. In the global validation, 5,430 associations are used to uncover potential associations. In the local validation, for a special disease, all known associations related to this special disease are reset as unknown associations. Then, other known associations are implemented to uncover potential associations. We extract top 50 candidate associations for each special disease. All predicted candidate associations are found in Supplementary Table 1. The statistical results are shown in Table 2. *GV* and *LV* are the numbers of confirmed associations in the top 50 by using global validation and local validation, respectively. *P*1 and *P*3 are the proportion of confirmed associations in the top 50 by using global validation and local validation, respectively. *D*1 is the number of miRNAs, and those miRNAs are associated with special disease and belonging to 498 miRNAs. The associations between those miRNAs and special disease can be verified from databases, like dbDEMC or miR2Disease. *P*2 is the proportion of *D*1 in the 495 miRNAs. *D*2 is the number of miRNAs, and those miRNAs are associated with special disease and belonging to 498 miRNAs. The associations between those miRNA and special disease can be verified from databases, like dbDEMC or miR2Disease or HMDD. *P*4 is the proportion of *D*2 in the 495 miRNAs. In Table 2, we find that *P*1 and *P*3 are significantly greater than *P*2 and *P*4 for the majority of diseases, respectively, excepting Biliary Tract Neoplasms and Skin Neoplasms. We also find that all candidate associations related with five diseases (Breast Neoplasms, Colorectal Carcinoma, Gastric Neoplasms, Pancreatic Neoplasms, and Lung Neoplasms) are confirmed for local validation. It demonstrates that MDA-SKF has excellent reliability for uncovering the associations between miRNAs and diseases.

**Table 2:** The results of global validation and local validation.

**Disease**	**Global validation**					**Local validation**				
	**GV^**a**^**	**P1(%)^**b**^**	**D1^**c**^**	**P2(%)^**d**^**	**Success^**i**^**	**LV^**e**^**	**P3(%)^**f**^**	**D2^**g**^**	**P4(%)^**h**^**	**Success^**i**^**
Adrenocortical carcinoma	11	22	69	13.94	**°**	27	54	99	20	**°**
Biliary tract neoplasms	2	4	136	27.47	**×**	6	12	144	29	**×**
Bladder neoplasms	32	64	95	19.19	**°**	39	78	126	25	**°**
Brain neoplasms	44	88	222	44.85	**°**	45	90	224	45	**°**
Breast neoplasms	43	86	394	79.60	**°**	50	100	433	87	**°**
Cervical neoplasms	37	74	108	21.82	**°**	38	76	109	22	**°**
Chordoma	16	32	59	11.92	**°**	19	38	59	12	**°**
Colon neoplasms	43	86	344	69.49	**°**	46	92	354	72	**°**
Colorectal carcinoma	45	90	385	77.78	**°**	50	100	425	86	**°**
Endometrial neoplasms	5	10	33	6.67	**°**	21	42	62	13	**°**
Esophageal neoplasms	39	78	262	52.93	**°**	43	86	277	56	**°**
Gastric neoplasms	42	84	342	69.09	**°**	50	100	401	81	**°**
Head and neck neoplasms	28	56	164	33.13	**°**	40	80	187	38	**°**
Hepatocellular carcinoma	43	86	326	65.86	**°**	49	98	326	66	**°**
Kidney neoplasms	44	88	314	63.43	**°**	43	86	316	64	**°**
Leukemia	44	88	243	49.09	**°**	46	92	243	49	**°**
Liver neoplasms	16	32	79	15.96	**°**	32	64	113	23	**°**
Lung neoplasms	45	90	376	75.96	**°**	50	100	394	80	**°**
Lymphoma	47	94	337	68.08	**°**	48	96	339	68	**°**
Melanoma	31	62	283	57.17	**°**	49	98	321	65	**°**
Mesothelioma	14	28	82	16.57	**°**	22	44	103	21	**°**
Nasopharyngeal neoplasms	33	66	272	54.95	**°**	38	76	276	56	**°**
Carcinoma, neuroendocrine	7	14	33	6.67	**°**	8	16	36	7	**°**
Carcinoma, oral	32	64	190	38.38	**°**	41	82	201	41	**°**
Ovarian neoplasms	33	66	299	60.40	**°**	48	96	340	69	**°**
Pancreatic neoplasms	43	86	388	78.38	**°**	50	100	397	80	**°**
Prostate neoplasms	45	90	296	59.80	**°**	49	98	339	68	**°**
Retinoblastoma	17	34	105	21.21	**°**	33	66	121	24	**°**
Sarcoma	40	80	206	41.62	**°**	38	76	206	42	**°**
Skin neoplasms	0	0	2	0.40	**×**	0	0	9	2	**×**
Testicular neoplasms	1	2	4	0.81	**°**	2	4	10	2	**°**
Thyroid neoplasms	23	46	115	23.23	**°**	33	66	140	28	**°**

a *GV is the number of confirmed associations in top 50 when using global validation*.

b *P1 is the proportion of GV in the top 50 associations*.

c *D1 is the number of miRNAs. Those miRNAs are associated with special disease and belonging to 498 miRNAs. The associations between those miRNAs and special disease can be verified from databases, like dbDEMC or miR2Disease*.

d *P2 is the proportion of D1 in the 495 miRNAs*.

e *LV is the number of confirmed associations in top 50 when using local validation*.

f *P3 is the proportion of LV in the top 50 associations*.

g *D2 is the number of miRNAs. Those miRNAs are associating with special disease and belonging to 498 miRNAs. The associations between those miRNAs and special disease can be verified from databases, like dbDEMC or miR2Disease or HMDD*.

h *P4 is the proportion of D2 in the 495 miRNAs*.

i *For global validation, It demonstrates that MDA-SKF gets excellent performance when P1 is larger than P2. For local validation, It demonstrates that MDA-SKF gets excellent performance when P3 is larger than P4. It is recorded as ° otherwise it is recorded as ×*.

To find some important miRNAs and potential associations, we analyze candidate associations relating with eight important human diseases (Breast Neoplasms, Colorectal Carcinoma, Gastric Neoplasms, Pancreatic Neoplasms, Lung Neoplasms, Colon Neoplasms, kidney neoplasms, lymphoma). Among them, six disease (Breast Neoplasms, Colorectal Carcinoma, Gastric Neoplasms, Pancreatic Neoplasms, Lung Neoplasms, Colon Neoplasms) are the top six diseases that are related to more miRNAs in the dbDEMC and miR2Disease database, and kidney neoplasms and lymphoma are used as case studies in many previous paper.

**Figure 7:**
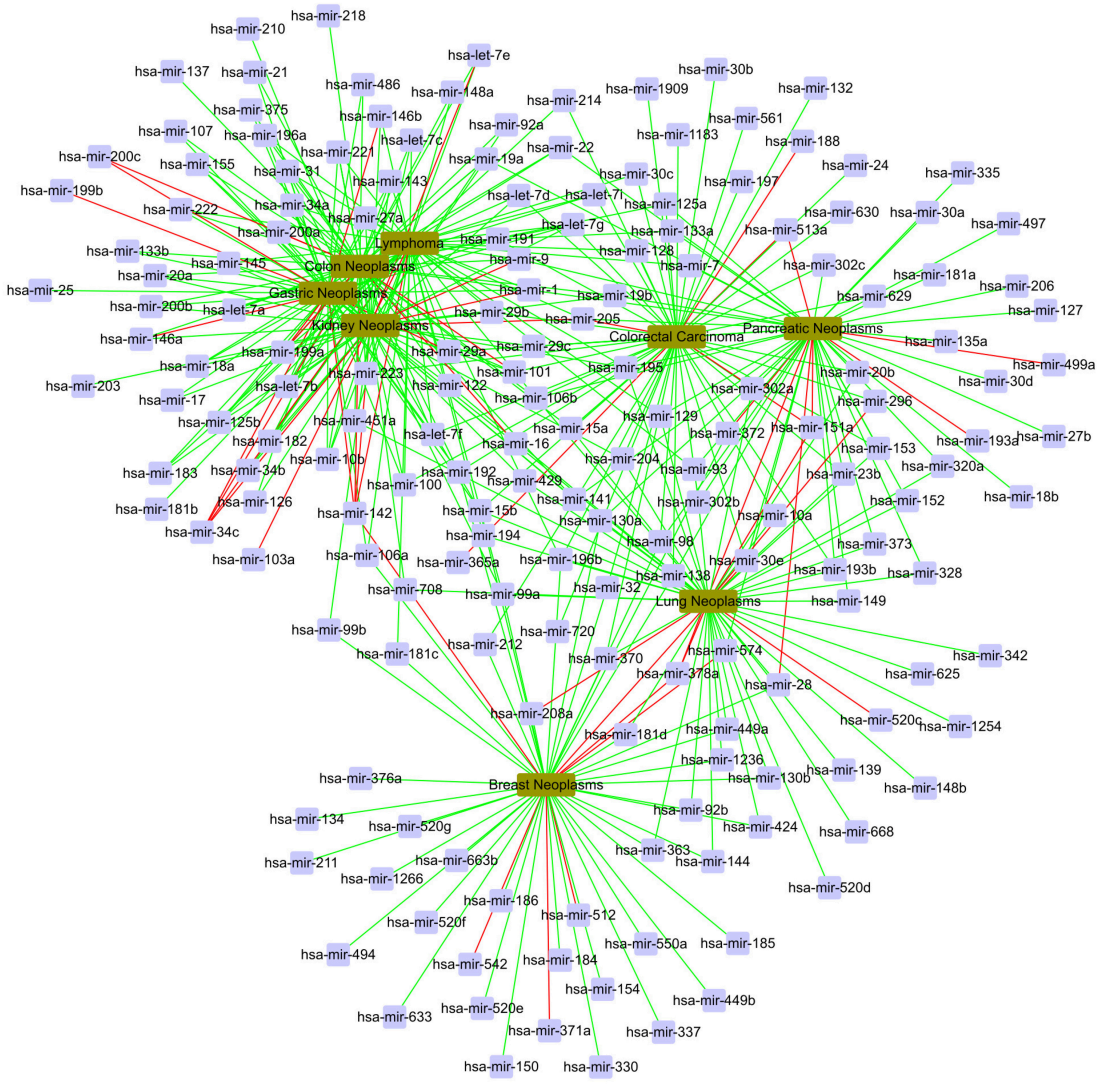
The case study in the global verification. The red line represents unconfirmed; the green line represents confirmed.

In the global validation, we gain a total of 400 candidate associations for eight diseases. The confirmed results are shown in Figure 7. In Figure 7, the red line represents unconfirmed and the green line represents confirmed. It can be find that most of candidate associations are confirmed by the miR2Disease and dbDEMC databases. It is obvious that five diseases are related to the same set of miRNAs, including hsa-let-7g, hsa-mir-1, hsa-mir-106b, hsa-mir-142, hsa-mir-15b, hsa-mir-223, and hsa-mir-29a.

**Figure 8:**
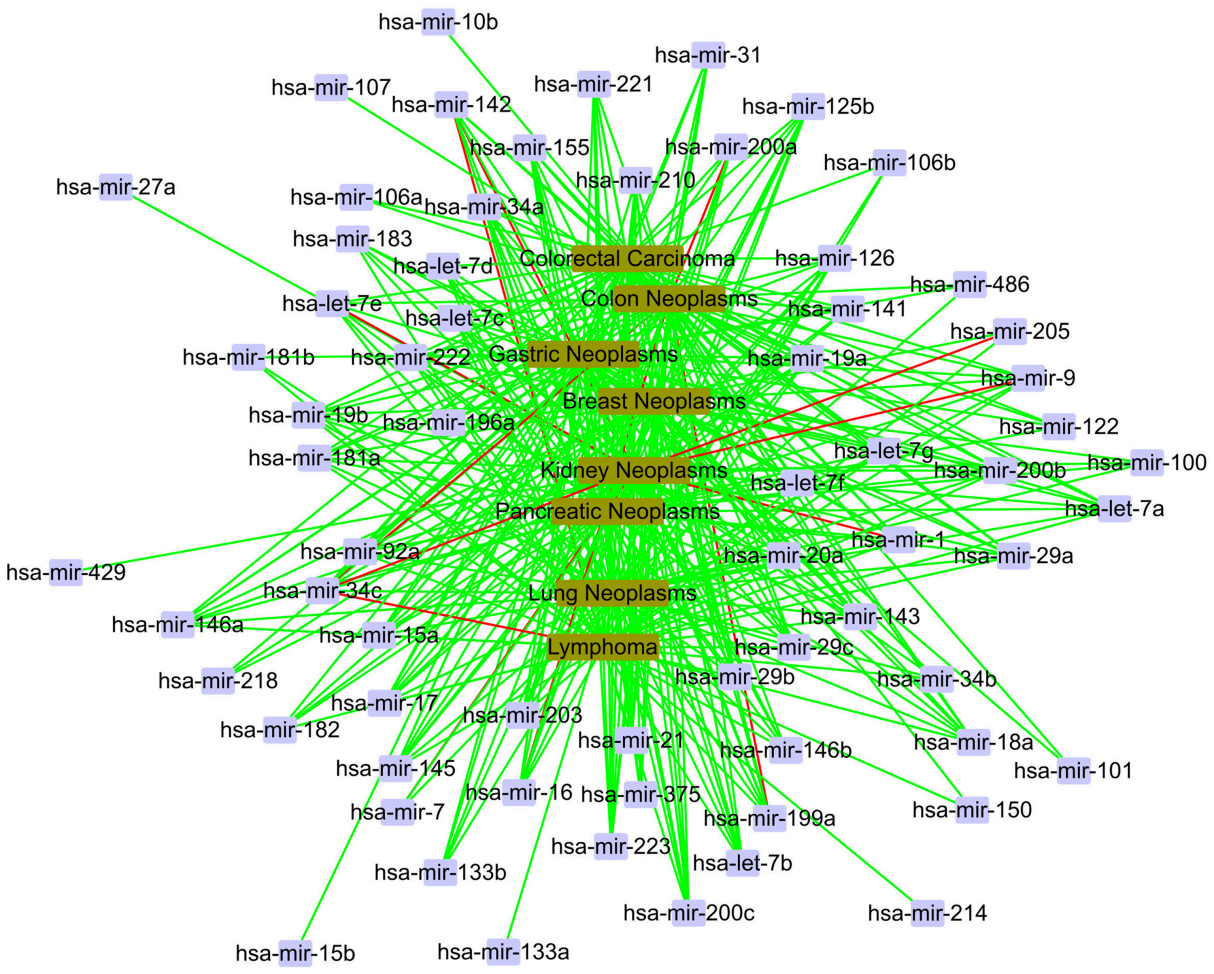
The case study in the local verification. The red line represents unconfirmed; the green line represents confirmed.

In the local validation, we also gain a total of 400 candidate associations for eight diseases. The confirmed results are shown as Figure 8. In Figure 8, we find that most of 400 candidate associations are confirmed by the HMDD, miR2Disease and dbDEMC databases. It is obvious that eight diseases are related to the same set of miRNAs, including hsa-let-7a, hsa-let-7b, hsa-mir-1, and so on. It is worth noting that three associations, hsa-mir-34c and kidney neoplasms, hsa-mir-34c and lymphoma, hsa-mir-34c and colon neoplasms, are unconfirmed in the current databases. Meanwhile, hsa-mir-34c is related to other five diseases in the database. Therefore, we believe that these three novel associations have a high probability of linkage between miRNAs and diseases, and they need more attention in subsequent traditional experiments.

## 4. Conclusions

We propose MDA-SKF to uncover potential miRNA-disease associations in the paper. First, we extract three miRNA kernels (miRNA functional similarity, miRNA sequence similarity, miRNA Hamming profile similarity kernel) and three disease kernels (disease semantic similarity, disease functional similarity, disease Hamming profile similarity kernel) to embody the similarity of miRNAs and diseases, respectively. Then, we propose Similarity Kernel Fusion (SKF) model by using original information of each kernel and the newly designed noise-reduction methods to better integrate multiple kernels. Then, Laplacian Regularized Least Squares (LapRLS) is employed on integrated kernels to uncover potential miRNA-disease associations.

Many experiments show that compared with other seven outstanding models, MDA-SKF has better precision on the three evaluation methods (global LOOCV, local LOOCV, and 5-fold CV). In order to further evaluate the reliable of MDA-SKF, two validation methods (global validation and local validation) are used to execute case studies of 32 diseases. A large number of candidate associations are confirmed by the HMDD, dbDEMC and miR2Disease databases. In addition, three associations (hsa-mir-34c and kidney neoplasms, hsa-mir-34c and lymphoma, hsa-mir-34c and colon neoplasms) and some special miRNAs (hsa-let-7g, hsa-mir-1, hsa-mir-106b, etc) need more attention. The future work may further take more machine learning methods and more similarity kernels into account to accurately uncover associations between miRNAs and diseases. Also, similar strategy can be applied in the other link prediction problems, such as circular RNA detection (Zeng et al., 2017b), disease gene prediction (Zeng et al., 2016a, 2017a) and sequence analysis (Zou et al., 2018).

## Data Availability Statement

The datasets and codes for this study can be found in the https://github.com/guofei-tju/MDA-SKF.

## Author Contributions

FG, YD, and LJ conceived and designed the experiments. LJ and YD performed the experiments and analyzed the data. FG and LJ wrote the paper. FG and JT supervised the experiments and reviewed the manuscript.

### Conflict of interest statement

The authors declare that the research was conducted in the absence of any commercial or financial relationships that could be construed as a potential conflict of interest.
